# Comparing two remote video survey methods for spatial predictions of the distribution and environmental niche suitability of demersal fishes

**DOI:** 10.1038/s41598-017-17946-2

**Published:** 2017-12-15

**Authors:** Ronen Galaiduk, Ben T. Radford, Shaun K. Wilson, Euan S. Harvey

**Affiliations:** 1 Department of Environment and Agriculture, Curtin University, Kent Street, Bentley, 6845 Australia; 2 Australian Institute of Marine Science, The University of Western Australia, 39 Fairway, Crawley, 6009 Australia; 3 The UWA Oceans Institute, The University of Western Australia, Fairway, Crawley 6009 Australia; 4 School of Earth and Environment, The University of Western Australia, 35 Stirling Highway, Crawley, 6009 Australia; 5 Marine Science Program, Science Division, Department of Biodiversity, Conservation and Attractions, Kensington, 6151 Australia

## Abstract

Information on habitat associations from survey data, combined with spatial modelling, allow the development of more refined species distribution modelling which may identify areas of high conservation/fisheries value and consequentially improve conservation efforts. Generalised additive models were used to model the probability of occurrence of six focal species after surveys that utilised two remote underwater video sampling methods (i.e. baited and towed video). Models developed for the towed video method had consistently better predictive performance for all but one study species although only three models had a good to fair fit, and the rest were poor fits, highlighting the challenges associated with modelling habitat associations of marine species in highly homogenous, low relief environments. Models based on baited video dataset regularly included large-scale measures of structural complexity, suggesting fish attraction to a single focus point by bait. Conversely, models based on the towed video data often incorporated small-scale measures of habitat complexity and were more likely to reflect true species-habitat relationships. The cost associated with use of the towed video systems for surveying low-relief seascapes was also relatively low providing additional support for considering this method for marine spatial ecological modelling.

## Introduction

Finfish are widely recognised as an important component of marine systems with crucial roles in terms of ecological processes, tourism, and fisheries^1,2^. Anthropogenic influences such as climate change, habitat loss and increased fishing pressure alter the biodiversity, abundance and distribution of finfish, potentially compromising their ecological roles and services^3,4^. However, the extent of these changes is not always apparent over spatial scales relevant to management. There is a need for new or improved tools to rapidly assess and predict significant species-environment patterns across varying spatial scales in a cost-effective manner.

Remote video systems provide a way to non-destructively survey fish assemblages at depths beyond the limits of SCUBA diving and are a common method for surveying patterns of assemblage composition and population dynamics of fish^5,6^. Models that pair video observations data of fish with benthic habitat data that has been remotely sensed using hydroacoustic or LiDAR technologies have become a powerful tool for understanding the relationships between demersal fish species and their environments e.g.^7–9^. Furthermore, pairing these species-distribution models (SDMs) with GIS and extrapolating models into non-surveyed areas has the potential to improve understanding of distributions in unsurveyed areas or how they may change in the future^10^. Among the various techniques currently available for remote video sampling of fish, baited remote video (BRUVs) are probably the most established^11^. BRUVs have been used to monitor individual species targeted by fisheries, fish assemblage composition^12,13^, the effectiveness of marine protected areas^14^ and the impact of seismic surveys and oil spills (http://www.aims.gov.au/docs/research/monitoring/seabed/video-monitoring.html, accessed March 2016). In recent years, data obtained from BRUVs has also been widely used in SDMs^6,15–17^. However, there are problems associated with this method that limit the precision and predictive power of the models.

Baited video systems attract fishes to a bait plume or camera station, and it is common practice to deploy the individual BRUVs systems at least 250–500 m apart to keep observations independent^18,19^. Hence the premise for spatial analysis for data collected with BRUVs is that this method collects information over a broad spatial range, the dimensions of which are dependent on local hydrology and sensory abilities of fish^6,20^. This could create a discrepancy when modelling small-scale species-habitat relationships from BRUVs data and reduce the accuracy of the ecological niche predicted by the model for each species. For example, in the study by^15^, sand-affiliated species were predicted to be present over reef probably due to an aggregation effect induced by baiting.

Small-scale landscape heterogeneity has ecological value, supporting different and diverse communities^21^ or key community processes such as distribution and abundance of prey or risk of predation^22^. At larger scales, landscape heterogeneity that considers combinations of both patchy and contiguous habitats is required to maximise fish diversity and abundance^7^. Thus, while models based on broad-scale habitat classification provide a good fit and predictive accuracy, fine-scale models explain a greater proportion of observed patterns in distribution and adopting a multiscale modelling approach can provide greater insight into spatial ecology of demersal fish^16^. Furthermore, environmental variables that are significant at coarse spatial scales may not be relevant at finer spatial resolution^16,23^. These studies highlight the importance of fine-scale habitat information when modelling species distributions and the potential biases that BRUVs can introduce.

Towed video systems have advantages similar to baited video systems, as they can be deployed at great depths, are non-destructive and provide a permanent record of fish lengths^11^. In addition, towed video produces comparable results to diver-operated video transects^24,25^ and is thought to be the least biased method for sampling abundance and biomass of sparids across multiple size ranges^2^. Additional benefits of the towed video are that they continuously capture data over seascape transition zones^26^. The transition zones between different benthic substrates have previously been identified as important determinants of fish assemblage structure and diversity^20^, because they provide a broader array of refuges and increased foraging and spawning opportunities^27^. Furthermore, towed video is a useful technique for rapid surveys of low-relief seascapes, vastly reducing manpower and vessel time^28,29^. Known limitations of towed video are typically associated with movement of the system through the water column. Fish that exhibit avoidance behaviour to moving objects could be frightened by the camera system which could result in low estimates of abundance and species richness^30^, while other species may be attracted to moving objects. Towed video may also get tangled and underestimate cryptic fish especially when the system is towed over highly rugose reef or dense macroalgal canopy and consequentially bias model predictions by including false absences^28,31^.

In this study, we compare fish species-environment relationships derived from either BRUVs or towed video systems (hereafter BV and TV, respectively) and use these to develop species distribution models. The specific aims of this study were (1) To model environmental niche requirements for fish and compare environmental variables from best-fit models between survey methods. (2) To develop predictive maps of fish distributions based on identified environmental niches and compare these predictions across two survey methods. (3) To assess cost-effectiveness of each method to facilitate decisions about which method is most suitable for SDMs.

## Results

### Model selection and variable contributions

The best models for explaining probabilities of occurrence differed between methods for all six fish species (Table 1). Occasionally there were several candidate models tied for best with none or only marginal differences in Akaike weights for evidence support (e.g. candidate models for *Eupetrichthys angustipes* BV in Supplementary Table S1). The explanatory power of the best models did not generally differ greatly between methods for the same species. Notable exceptions were models for *Coris auricularis* using BV data, which had higher adjusted R^2^ values than models using TV data, and vice versa for *Eupetrichthys angustipes* (Table 1).

**Table 1:** GAMs of best fit for predicting probability of occurrence of the six study species across two survey methods: baited video (BV) and towed video (TV).

Species/method	Intercept	Bathymetry	Slope	Curvature	Plan	Profile	Range10	Range2	Range5	Eastness	Adjusted R^2^	df	AICc	∆AICc	Akaike weight
*Austrolabrus maculatus* BV	0.026	+									0.06	3	199.43	0	0.12
*Austrolabrus maculatus* TV	−0.018	+	+			+		+			0.15	9	242.38	0	0.06
*Coris auricularis* BV	1.132	+					+	+			0.29	7	155.27	0	0.22
*Coris auricularis* TV	0.016	+							+		0.11	5	465.88	0	0.13
*Eupetrichthys angustipes* BV	−0.938								+		0.06	3	180.93	0	0.05
*Eupetrichthys angustipes* TV	−0.433	+									0.43	3	94.54	0	0.26
*Notolabrus parilus* BV	0.670	+									0.13	3	176.57	0	0.14
*Notolabrus parilus* TV	0.153		+								0.11	3	142.67	0	0.12
*Ophthalmolepis lineolatus* BV	1.154	+					+				0.22	5	164.41	0	0.20
*Ophthalmolepis lineolatus* TV	−0.219	+									0.09	3	226.77	0	0.16
*Upeneichthys vlamingii* BV	0.268					+					0.02	3	202.32	0	0.10
*Upeneichthys vlamingii* TV	−0.067	+								+	0.1	5	177.95	0	0.11

Best descriptor variables identified by (+). Full summary of candidate models (∆AICc < 2) is presented in Supplementary Table S1.

**Figure 1:**
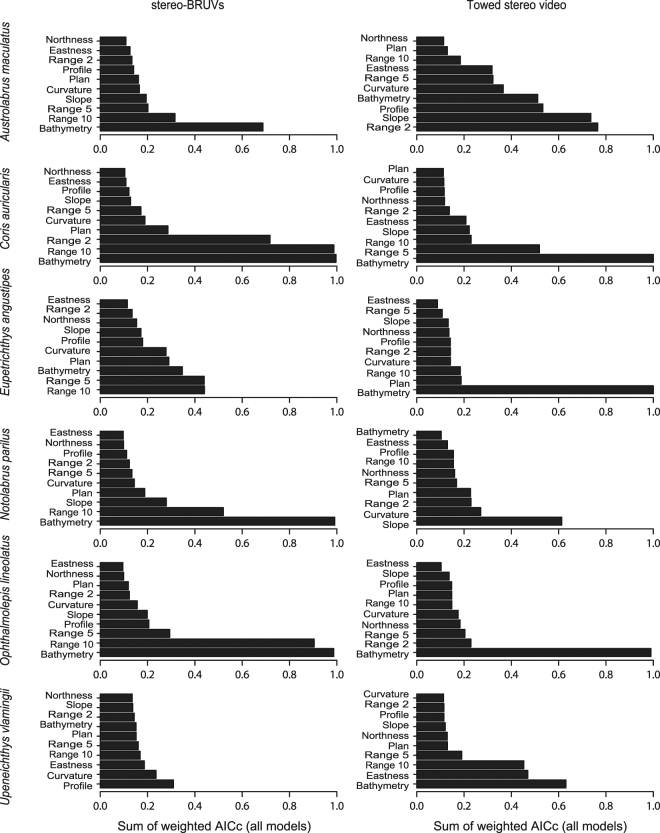
Relative importance of all fitted environmental variables as indicated by the sum of weighted AICc for each variable across all fitted models.

The most important variables for explaining the probability of occurrence of the study species across two survey methods was bathymetry followed by the range variable, which is indicative of structural complexity or relief (Fig. 1 and Table 1). The bathymetry variable was consistently identified as important with exception being models fitted for *Notolabrus parilus* when using the TV method and *Upeneichthys vlamingii* when using the BV method. Indeed all models for *U. vlamingii* presence derived from BV data were generally poor accounting for ≤2% variance in data. Range was also consistently included in models, though the spatial scale at which relief was considered important varied among species and methods. When using TV data, a finer scale relief (range 2) was often considered more important than broader spatial measures of relief (range 10). Conversely, models using BV data consistently included range 10 as an important variable (Fig. 1, Supplementary Table S1).

There were linear and non-linear correlations between the environmental variables and probability of occurrence of all study species identified by the GAMs of best fit (Supplementary Fig. S1). Nine out of twelve models of best fit had bathymetry as important environmental variable. The probability of occurrence of all species was typically higher in deeper water with exception to *Notolabrus parilus* when using the BV method. Range 10 and slope both positively correlated with probabilities of species’ occurrence, while range 5 had mixed effect on probabilities of occurrence. Range 2, profile (concavity/convexity of the slope) and easting (azimuthal slope direction) all had linear negative correlations with probabilities of occurrence of the study species (Supplementary Fig. S1).

### Predictive performance

The predictive performance of models of best fit developed for the six species, was good for one model (AUC 0.8–0.9), fair for two models (AUC 0.7–0.8), and poor for nine models (0.5 < AUC < 0.7; Table 2). Models developed for the TV method had consistently better predictive performance, the exception being for *Ophthalmolepis lineolatus* models where the BV method had a slightly higher AUC. Similar general trends were evident for Kappa statistics, with models developed for the TV method having greater Kappa values except the *O. lineolatus* BV model (Table 2). Sensitivity values (correct presences) ranged from 0.41 to 0.75 and specificity ranged from 0.48 to 0.78 (correct absences). The total proportion of correct predictions (presence and absence) ranged from 0.44 for *Upeneichthys vlamingii* BV to 0.77 for *Coris auricularis* TV (Table 2).

**Table 2:** Summary of model predictive performance for each fish species across two survey methods: baited video (BV) and towed video (TV).

Species/method	*P*_fair_ threshold for presence	Proportion Correctly Classified	Sensitivity	Specificity	Kappa	AUC
*Austrolabrus maculatus* BV	0.54	0.62	0.65	0.61	0.24	0.64
*Austrolabrus maculatus* TV	0.5	0.67	0.67	0.68	0.34	0.66
*Coris auricularis* BV	0.6	0.7	0.7	0.71	0.35	0.74
*Coris auricularis* TV	0.48	0.77	0.75	0.78	0.54	0.82
*Eupetrichthys angustipes* BV	0.33	0.5	0.5	0.5	0	0.61
*Eupetrichthys angustipes* TV	0.52	0.69	0.68	0.7	0.36	0.68
*Notolabrus parilus* BV	0.71	0.51	0.51	0.5	0.01	0.51
*Notolabrus parilus* TV	0.48	0.54	0.56	0.53	0.09	0.6
*Ophthalmolepis lineolatus* BV	0.66	0.7	0.7	0.7	0.37	0.76
*Ophthalmolepis lineolatus* TV	0.5	0.58	0.58	0.57	0.15	0.62
*Upeneichthys vlamingii* BV	0.57	0.44	0.41	0.48	−0.1	0.57
*Upeneichthys vlamingii* TV	0.54	0.52	0.52	0.53	0.05	0.62

Presences and absences for assessing sensitivity and specificity were determined using *P*_fair_ as threshold.

### Mapping species distributions

**Figure 2:**
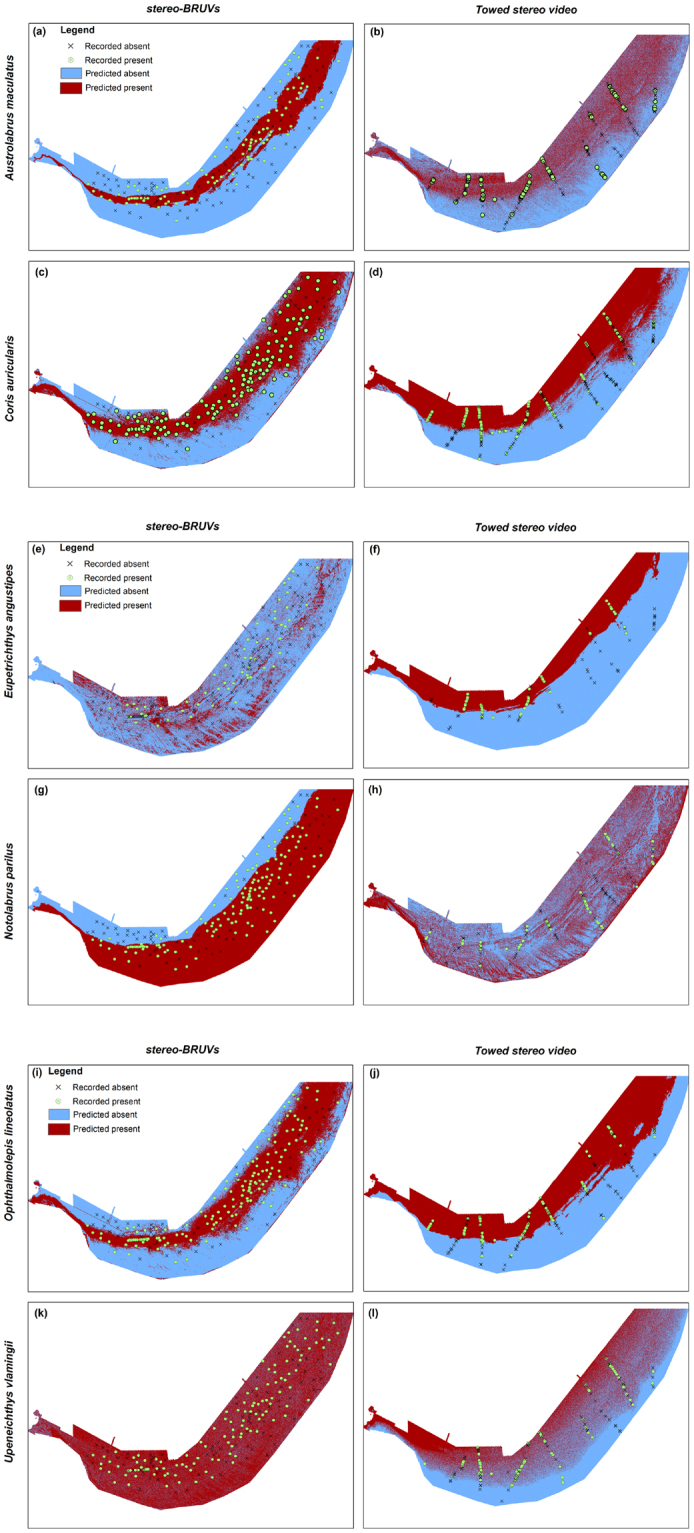
Predicted niche distributions in Geographe Bay as defined by the GAMs of best fit for individual study species across two sampling methods.

Presence absence maps provided a detailed representation of continuous predicted distributions of the six species using the two survey methods (Fig. 2 and Supplementary Fig. S1 for partial response plots as result of GAMs of best fit). The distribution of *Austrolabrus maculatus*, *Coris auricularis* and *Ophthalmolepis lineolatus*, all reef associated species, were predicted to be in close proximity to the reef ridge by both survey methods (Fig. 2a–d,i,j). In contrast, the ecological niche predictions for *Eupetrichthys angustipes*, *Notolabrus parilus* and *Upeneichthys vlamingii* differed between the two survey methods. The best fit GAM for *E. angustipes* from the BV data predicted this species to be spread across the bay and associated with flat areas. Whereas habitat associations predicted by the TV data for this species were mainly in deeper waters (Fig. 2e,f). The distribution of *Notolabrus parilus* using the BV data predicted high probability of detection along the shallow reef ridge. Whereas the distribution based on the TV data, predicted this species to more closely associate with steep terrain and maps show an even distribution across much of the bay with high probability of detection in the more exposed western part of the bay (Fig. 2g,h). Models for explaining variation in *Upeneichthys vlamingii* presence using BV data had weak explanatory power (Table 1) and there was no particular area of the bay that was recognised unsuitable for the *Upeneichthys vlamingii* based on BV data. Predictions from the TV data however mapped intermediate to deep water areas as most suitable for this species (Fig. 2k,l).

### Costs

The main difference in the costs associated with the two methods relate to general logistics and pre-field preparations. Surveys using BV method require a vessel large enough to deploy 10 video systems and accommodate an additional crew member compared to the smaller boat and crew required to deploy a single towed video system. Consequently, the vessel and camera systems associated costs could be as much as 6 to 7 times higher for surveys performed with BRUVs. In addition, pre-field system calibrations took an extra five hours for the BV method (Table 3).

**Table 3:** General costs and staff time budgets (total hours devoted to each activity) associated with data collection by each of the survey methods.

	Baited video	Towed video
*General logistics*		
Vessel costs ($AU/day)	2000^a^	350^a^
Camera system costs($AU/day)	2000^b^	400^b^
*Pre-Field*		
Equipment calibration and processing (staff hours)	8	3
*In-Field*		
Data collection (total)	132^c^	136^c^
Video download	2	0.5
*Post-field*		
Video processing total	1 h video recording = 3 h processing	1 h video recording = 3 h processing

^a^Large vessel carrying 4 crew and staff deploying 10 BRUVs; small vessel carrying 3 crew and staff deploying one towed video system. ^b^Calculations based on 10 BRUVs and one towed video systems. ^c^BRUVs = 3 staff × 5.5 days × 8 h/day; tow ed video = 2 staff × 8.5 days × 8 h/day.

## Discussion

A combination of TV and, rarely utilised in marine studies, LiDAR hydroacoustic surveys is a robust, non-intrusive, low cost method for examining fine-scale species-environment associations, compared to BV, at least in environments with low structural complexity. Our results indicate that the choice of data collection method is important for fitting and performance of species distribution models. All fitted models for the TV method, except for the Southern Maori wrasse *Ophthalmolepis lineolatus*, provided a better model fit and had higher AUC values. This is probably due to TV introducing less variation in datasets by sampling fish in their natural habitat. While demonstrating the advantages of the TV approach, our findings do not preclude the use of BV for predictive modelling in the absence of TV capacity. Spatial modellers, however, should be aware that BV may lure fish from their natural habitat, thus introducing more variation into observed species-environment relationships e.g.^15^.

It is clear, however, that modelling species distributions over low relief, highly homogenous environments is challenging. The explanatory power and reduced model fits presented here are relatively low compared to models developed for a structurally complex, highly heterogeneous ecosystems such as coral or temperate reefs which utilised similar environmental variables^15,32^. All model fits from our study would benefit from incorporation of additional biotic variables, such as extent of canopy cover of macrophytes or occurrence of sessile invertebrates. For example, previously fitted GAMs for probability of occurrence of the Brown-spotted wrasse *Notolabrus parilus* and *O. lineolatus* using macroalgal type and presence of sessile biota among other substrate associated explanatory variables, were characterised by good model fits and AUC > 0.8 for data collected with BV^16^. In addition^33^, produced a much lower AUC value for the TV method than that reported here for Blue-spotted Goatfish *Upeneichthys vlamingii* when using only seafloor variables and a similar number of occurrences. However, the AUC value for GAM fitted for *U. vlamingii* from the BV method was much higher in the study by^33^. One possible explanation for this dissimilarity with our findings could be differences in prevalence of modelled species between the two studies. Prevalence of species is known to affect modelling outcomes and performance of models^34^. The overall sample sizes in both studies were similar, however the prevalence of *U. vlamingii* in our study was four times higher than that reported by^33^.

While the predictive performance of models varied between methods, the predicted distributions of species across the bay and the extent of the ecological niches predicted by both methods were similar for four of the study species. For the remaining two species (*Eupetrichthys angustipes* and *Upeneichthys vlamingii*), the distribution patterns were more clearly defined by the TV method. The similarity in niche predictions between the two datasets could be attributed to choice of the modelled species, which are mostly narrow distributional range and/or small size species. Small sized fish tend to have smaller home ranges and are less likely to move as far as larger bodied counterparts^35^. Furthermore, narrowly distributed species exhibit minimal niche variation, and are more reliably modelled when extrapolating to unsurveyed areas^28,36^. While the TV may provide more refined distribution models than BV, the applicability of higher resolution information to spatial management will most certainly vary among species in question. For example, large mobile carnivores would be better surveyed using BV, where bait is necessary for attracting these rarely occurring species into the camera system field of view^37^, or where the species of interest are scared by the TV camera system moving through the water. In addition, fish species associated with structurally complex habitats or cave-dwelling species may be more effectively surveyed using methods that can effectively search caves and overhangs^29,38,39^. However, where there is extensive low-relief habitat, such as the seagrass meadows surveyed by this study, the TV appears to perform better than the BV in terms of examining the natural relationships between fish and their habitat. Moreover, models based on TV datasets that utilise stereo-video capabilities, allow boundaries of a surveyed area to be defined and absolute species abundance or density can be calculated, and are a significant step towards improving the biological appeal of spatial modelling in the marine environment^20,40,41^.

We found that bathymetry was a good predictor of occurrence patterns of endemic fish species, though the relative importance of depth differed among species and with survey methods. Fish diversity and abundance is often distributed along a depth gradient with many species only occurring within certain depth ranges^42,43^. This may be because depth can be a proxy for other environmental variables, such as light penetration, which influences the distribution and species composition of seagrass and algae^44^, which are prominent in the survey area. Canopy-forming seaweeds can drive distribution patterns of fish species that rely on these habitats for food^45^, shelter^46,47^ or nesting^48^.

The spatial scale at which structural complexity was measured was also an important predictor of fish occurrence and differed among species. This may relate to different sized species requiring different sized refuges^27,49,50^, interspecific variation in motility and home range^35^, or the extent of habitat specialisation^51,52^. However, the regular inclusion of large-scale structural complexity of habitat from the BV most probably related to fish being attracted from surrounding habitats to a single focus point by the bait. Conversely, models based on the TV data often incorporated complexity measured across a smaller scale, reflecting the movement of the system across the seascape and recording fish presence in areas they inherently occupy and use as refuge within their normal home range. Clearly, depth and structural complexity are good predictors of fish distributions, and as these metrics are also indicative of key processes that relate to resilience in other systems^53^ they are important variables for spatial planning of marine reserves. Moreover, maintaining connectivity between habitat patches with different levels of complexity across seascape maintains the structure of fish communities and ecosystem function^54,55^.

The lower survey cost associated with the use of TV compared to the BV provides additional support for considering this method for marine spatial management purposes. The level of expertise and time required for collecting and processing data from the two methods is virtually identical, the major difference being costs associated with vessel hire and the purchase of camera systems. The initial outlay of purchasing equipment is also five times greater when using BV, though repeated use of the same cameras would reduce the long term differences. The daily costs associated with needing a larger vessel and extra crew will, however, become more relevant on longer field trips. Where possible, a combination of both survey methods will provide greater insight and confidence into assemblage and species distributions for applied management purposes such as conservation spatial planning.

In conclusion, research programs must choose survey techniques and indicators applicable to their research questions^29,37^. While BRUVs are a well-established method for surveying fish assemblages, their usefulness for species distribution modelling should be revised due to the biases they may introduce with respect to habitat associations of fish. Other methods for surveying fine-scale species-habitat associations typically involve divers (DOVs, underwater visual census), and are limited by diving depths and times. Video from towed or autonomous underwater vehicles are, however, less constrained by depth and could become an effective method that combines the benefits of a remote video and a fine spatial scale observations of species-habitat associations. Our study provides evidence that towed video is a robust, non-intrusive, low cost method for fine-scale data collection that can be useful for spatial ecological modelling of mobile biota such as demersal fish. In combination with precise habitat data from remote sensing systems such as LiDAR, developments in towed video methods such as stereo-video capacity can map demersal species distributions as well as sessile biota and may allow rapid identification of sensitive or ecologically significant areas which are important for marine conservation.

## Methods

### Study area

Geographe Bay is a ~100 km wide, relatively shallow, north-facing embayment with seagrass cover that can at times exceed 60%^56^. The bay is located in southwestern Australia, approximately 220 km south of Perth (Supplementary Fig. S2). The majority of the seafloor is covered by unconsolidated sediments that have been deposited over older clay layers. There is also a series of discontinuous limestone ridges of varying height profiles (from <1 m to approx. 2.5 m), dominated by canopy-forming brown macroalgae, that run parallel to the coast^57,58^.

### Fish occurrence data

Fish occurrence data was collected between the 9^th^ and17^th^ December 2014. This research was conducted in accordance with all relevant guidelines and regulations following permits AEC_2014_21 and SF009757 issued by the Curtin Animal Ethics Committee and WA Department of Parks and Wildlife respectively. Two methods were used for sampling fish assemblages in Geographe Bay: a point observation method using BV and a transect method using TV. The BV sampling was spatially stratified according to the size of the study area and depth: random points for sampling were allocated to adequately cover the bathymetric gradient in the bay, although major substrate types (e.g. reef ridge) were particularly targeted based on the skipper’s local knowledge of the study area. To avoid bait plume overlap and reduce the likelihood of fish moving between BV systems, systems were at least 400 m apart from each other. Each system comprised two wide-angle Sony CX12 high-definition video cameras that had been baited with approximately 800 g of crushed pilchards (*Sardinops sagax*), and lowered to the bottom for a 60 minute soak time. The 217 video recordings from these deployments were analysed using the software EventMeasure (SeaGIS Pty Ltd). We only included fish within seven metres of the front, 2.5 m on each side of the cameras and approximately 3 m into the water column above the BV system. Additional information on design, calibration^59^ and use of the BRUVs is presented in detail in the literature e.g.^18,60^ and references threin.

The TV camera system also consisted of two wide angle Sony CX12 high-definition video cameras mounted 0.7 m apart on a custom built cage to protect the system during collisions and provide a secure towing point. The cameras were inwardly converged at eight degrees to gain an optimized field of view with stereo-coverage from 0.5 m to the maximum visibility. The same distances for fish inclusion in the video as for the BV were applied for this method. The system was towed off the front of a 4.6 m vessel at speeds 0.5–2 knots at a height approximately 1 m above the seafloor and tilted downward to cover the immediate benthos as well as the water column in the field of view of the cameras. This configuration facilitated an ample field of view to observe demersal fish allowing safe navigation of the equipment. Using the underwater viewer and the depth sounder, the camera system was positioned immediately below the centre of the boat to within 2 m behind the vessel. The video signals were transferred to the surface via an umbilical cable where they were monitored in real-time, time stamped and synchronised with positional data that were recorded at 2 s intervals with handheld GPS located in the centre of the boat. Nine video transects were executed capturing fish assemblage composition along continuous physical and biological gradients within the study area (e.g. substrate and benthic biological habitat). This amounted to 22 hours of georeferenced underwater towed video footage, covering 83 linear km of seafloor. Subsequently, the video footage of fish species occurrence were visually analysed with the EventMeasure software (www.seagis.com.au).

In both fish occurrence datasets, all fish were identified to the lowest taxonomic level possible and provided reliable species presence records for future modelling. As prevalence of species can affect modelling outcomes and performance of models^34^, we chose a subset of six focal species (five of which are limited range endemic species) that were often observed in both video survey techniques and represent a diversity of demersal fish life histories, size, and mobility in the study region (Supplementary Table S2). To generate pseudo-absences for the BV fish occurrence dataset, we assigned absence to each individual deployment where the particular focal fish taxon was not observed. This method has been previously used in modelling species-environment relationships^15,33^. The final presence-absence BV dataset was partitioned into training (75%) and testing (25%) data for individual focal species.

To generate reliable pseudo-absences for fish observations obtained from constantly moving TV system, we applied kernel density function to the focal species occurrence dataset using ArcGIS 10.2.2. The probability density function relies on assumption that presence is a probabilistic function mainly affected by species abundance and detectability^61,62^. Kernel density function was applied to point data with observed presences of the focal species to generate a continuous surface of probabilities of occurrence of the focal species along a transect. The neighbourhood search radius for kernel density calculations was set to 400 m to represent similar distance that was used for the BV systems. The results of probability surface were further analysed in PresenceAbsence package^63^ using R statistical software version 3.2^64^ in order to calculate the optimal threshold for translating a probability surface into presence-absence maps. We selected the optimal threshold based on the maximum values of Kappa, which is a commonly used chance-corrected measure of agreement for presence-absence ecological data^63,65^. The kernel density values below the optimal threshold were converted to pseudo-absences and true observations of focal species in the video recording from the TV system were kept as presences. The final pseudo-absences for modelling were randomly generated from combination of areas with kernel density below the appointed threshold and with no fish taxa observations from the TV to create a final ratio of 1:1 of true presences and pseudo-absences of a focal species along transects. The final presence-absence TV datasets were partitioned into training (75%) and testing (25%) data for individual modelled species.

### Habitat data

The bathymetric data was extracted from a mosaic of LiDAR and multibeam surveys collected by Fugro Corporation Pty Ltd gridded to a cell size of 4 * 4 m. The LiDAR hydrographic survey was performed between April and May 2009 on behalf of the Department of Planning as a part of a national coastal vulnerability assessment. The LiDAR area extended seaward from the coastal waterline to the 20 m marine nautical navigation chart contour and constituted the majority of bathymetric data. For further information on LiDAR collection and processing see www.planning.wa.gov.au, accessed July 2016. In addition to the LiDAR, a small area of deeper water was surveyed during March-April 2006 using Reson 8101 multibeam in the north-west part of the study area as part of the Marine Futures biodiversity surveys (see Radford *et al*. 2008 and matrix-prod.its.uwa.edu.au/marinefutures/research/project; accessed July 2016 for further details). Bathymetry data and the Spatial Analyst toolkit in ArcGIS 10.2.2 was used to derive nine variables that describe the structure and complexity of the seafloor and which have previously been shown to influence the distribution of fish^9,15^ (Supplementary Table S3).

### Species distribution modelling

To infer the effect of environmental variables (Supplementary Table S3) on the probability of occurrence of six fish taxa across the two survey methods, we applied generalised additive models (GAMs) developed for individual study species and the full subsets approach^66^. GAMs are the most common and well developed method for modelling fish habitats^67^ and the full subsets method provides an unconstrained approach for fitting ecological responses to the predictor variable^66,68^. All models were fitted with binomial error distributions and logit link functions in R version 3.2.0^64^. To produce conservative models and to avoid model overfitting, the number of smooths (knots) was restricted to k = 4^69^ and the model fits for all possible combinations of variables (total possible model fits = 1023) were compared using differences in Akaike Information Criterion corrected (**∆**AICc) for finite sample size^70^. In addition, to rank the fitted models we computed the Akaike weights^71^ to examine the weight of likelihood in favour of a model being the best in the given set of models. Best models were selected based on having lowest AICc value, smallest AICc difference (**∆**AICc < 2) and having the highest weight across all possible models^70^. To explore the relative importance of each variable, we summed the weighted AICc values across all possible models.

### Model evaluation and predictions

The test dataset was used to evaluate the discrimination and accuracy of the best developed models for all species across two methods. We used threshold independent Receiver Operating Characteristic (ROC) and the area under the curve (AUC) as graphical means to test the sensitivity (true positive rate) and specificity (false positive rate) of a model output^72,73^. The area under the ROC curve is a measure of overall fit and commonly varies between 0.5 (no predictive ability) and 1(perfect fit)^65^. In addition, we calculated a threshold dependent Kappa statistic which is commonly used in ecological studies with presence-absence data and provides an index that considers both omission and commission errors^65,74^. *P*_fair_ was chosen as the threshold to convert predicted probabilities of occurrence to presence/absence values as it minimises the difference between sensitivity and specificity and provides a measure of how well the model predicts both presences and absences^15,16^. *P*_fair_ was also found to be better at selecting a threshold value when the prevalence of species was not close to 50%^75^, as in the case of this study. Final comparison for model predictive performance across two survey methods were done by comparing the AUC values of best model fits developed for individual species.

Semivariograms were used to assess the level of spatial autocorrelation in the residuals of all models using Automap package in R^76^. Low levels of spatial autocorrelation (semi-variance 0.18–0.28) were found in TV datasets, which can be attributed to the initial method of generating pseudo-absences for this dataset. The kernel density function is relying on point observation of presences in order to generate continuous surfaces of probabilities of occurrence, which in turn were used to generate pseudo-absences. Furthermore, we plotted model residuals and final model predictions against the spatial coordinates to examine systematic spatial patterns in fitted models and distribution of correct/incorrect classifications. After evaluation, the best models for individual species were predicted on 4 * 4 grid using both train and test datasets across two sampling methods. Binary presence-absence maps were then constructed using the *P*_fair_ probability thresholds.

### Costs

Accurate time budgets were maintained for all activities associated with each methodology and were expressed in staff time (number of hours per person devoted to each activity;^38,77^). We also included direct costs associated with general logistics (e.g. vessel and camera systems cost) for each survey method. Time not directly associated with the actual survey task (e.g. travel time to and from survey sites, accommodation costs) was excluded as it was similar for both methods. Time budgets were divided into three categories: Pre-Field Time (e.g. equipment calibration: 10* BRUV systems, one towed video system), In-Field Time (e.g. data collection, video download), and Post-Field Time (e.g. video analysis). To make comparison possible, all estimates of In-Field costs were standardised to 40 * 60 minutes BRUV deployments (10 BRUV systems rotated four times within an eight hour day) and 8 hour-long video recording from the towed video system.

### Data Availability

The datasets analysed during the current study are available from the corresponding author on reasonable request.

## Electronic supplementary material

Supplementary Information 
