# Supplementary material for: Comparing two remote video survey methods for spatial predictions of the distribution and environmental niche suitability of demersal fishes
Source: Sci Rep. 2017 Dec 15;7:17633. doi: 10.1038/s41598-017-17946-2 (PMC5732166; doi:10.1038/s41598-017-17946-2)
Supplement: Supplementary file 1 — Supplementary Information [file 41598_2017_17946_MOESM1_ESM.pdf]

Supplementary information

Comparing two remote video survey methods for spatial predictions of the distribution and environmental niche suitability of demersal fishes

Ronen Galaiduk[1,2,*], Ben T. Radford[2,3,4], Shaun K. Wilson[3,5], Euan S. Harvey[1]

[1]Department of Environment and Agriculture, Curtin University, Kent Street, Bentley 6845, Australia

[2]Australian Institute of Marine Science, The University of Western Australia, 39 Fairway, Crawley 6009, Australia

[3]The UWA Oceans Institute, The University of Western Australia, Fairway, Crawley 6009, Australia

[4]School of Earth and Environment, The University of Western Australia, 35 Stirling Highway, Crawley 6009, Australia

[5]Marine Science Program, Science Division, Department of Environment and Conservation, Kensington, 6151, WA, Australia

[*]email: r.galaiduk@aims.gov.au

Supplementary Table S1. Best descriptor variables (+) and the summary of candidate models (ΔAICc < 2) for predicting probability of occurrence of the six study species across two survey methods: baited video (BV) and towed video (TV). GAMs of best fit identified by ΔAICc = 0 and highest Akaike weights for evidence support.

Species/method	Intercept	Bathymetry	Slope	Curvature	Plan	Profile	Range10	Range2	Range5	Eastness	Adjusted R^2	df	AICc	ΔAICc	Akaike weight
Australabrus maculatus BV	0.026	+									0.06	3	199.43	0	0.12
Australabrus maculatus BV	0.033	+					+				0.08	5	200.70	1.27	0.06
Australabrus maculatus TV	-0.018	+	+			+		+			0.15	9	242.38	0	0.06
Australabrus maculatus TV	-0.018		+			+		+			0.12	7	242.74	0.36	0.05
Australabrus maculatus TV	-0.016	+	+	+				+			0.14	9	243.19	0.80	0.04
Australabrus maculatus TV	-0.019		+			+		+		+	0.14	9	243.61	1.23	0.03
Australabrus maculatus TV	-0.014		+	+				+			0.11	7	243.70	1.32	0.03
Australabrus maculatus TV	-0.021	+	+			+		+		+	0.17	11	243.80	1.42	0.03

Austrolabrus maculatus TV	-0.016	+	+			+		+	+	0.17	11	243.86	1.48	0.03
Coris auricularis BV	1.132	+					+	+		0.29	7	155.27	0	0.22
Coris auricularis BV	1.169	+			+		+	+		0.32	9	156.58	1.31	0.11
Coris auricularis TV	0.016	+							+	0.11	5	465.88	0	0.13
Coris auricularis TV	0.015	+								0.1	3	466.33	0.45	0.11
Coris auricularis TV	0.016	+					+			0.11	5	467.29	1.41	0.07
Coris auricularis TV	0.018	+	+						+	0.12	7	467.40	1.52	0.06
Eupetrichthys angustipes BV	-0.938								+	0.06	3	180.93	0	0.05
Eupetrichthys angustipes BV	-0.930						+			0.06	3	181.04	0.11	0.05
Eupetrichthys angustipes BV	-0.981			+					+	0.09	5	181.61	0.68	0.04
Eupetrichthys angustipes BV	-0.964				+				+	0.09	5	181.65	0.72	0.03
Eupetrichthys angustipes BV	-0.961				+		+			0.09	5	181.66	0.73	0.03
Eupetrichthys angustipes BV	-0.971			+			+			0.09	5	181.77	0.84	0.03

Eupetrichthys angustipes BV	-1.004	+						+	0.08	5	182.21	1.27	0.03
Eupetrichthys angustipes BV	-0.999	+					+		0.08	5	182.30	1.37	0.03
Eupetrichthys angustipes BV	-1.038	+			+		+		0.12	7	182.63	1.70	0.02
Eupetrichthys angustipes BV	-1.036	+			+			+	0.12	7	182.70	1.77	0.02
Eupetrichthys angustipes BV	-1.047	+		+				+	0.12	7	182.83	1.90	0.02
Eupetrichthys angustipes BV	-0.975					+		+	0.08	5	182.86	1.93	0.02
Eupetrichthys angustipes TV	-0.433	+							0.43	3	94.54	0	0.26
Notolabrus parilus BV	0.670	+							0.13	3	176.57	0	0.14
Notolabrus parilus BV	0.729	+					+		0.16	5	176.87	0.30	0.12
Notolabrus parilus BV	0.865	+	+				+		0.2	7	177.39	0.82	0.09
Notolabrus parilus TV	0.153		+						0.11	3	142.67	0	0.12
Notolabrus parilus TV	0.203		+	+					0.15	5	143.96	1.28	0.06
Notolabrus parilus TV	0.194		+		+				0.14	5	144.23	1.56	0.05

Ophthalmolepis lineolatus BV	1.154	+			+				0.22	5	164.41	0	0.20
Ophthalmolepis lineolatus BV	1.191	+			+		+		0.25	7	165.79	1.38	0.10
Ophthalmolepis lineolatus TV	-0.219	+							0.09	3	226.77	0	0.16
Ophthalmolepis lineolatus TV	-0.232	+				+			0.11	5	228.35	1.58	0.07
Ophthalmolepis lineolatus TV	-0.223	+					+		0.11	5	228.59	1.82	0.06
Upeneichthys vlamingii BV	0.268			+					0.02	3	202.32	0	0.10
Upeneichthys vlamingii BV	0.267		+						0.02	3	203.04	0.73	0.07
Upeneichthys vlamingii BV	0.265							+	0.01	3	203.76	1.44	0.05
Upeneichthys vlamingii BV	0.264				+				0.01	3	204.02	1.70	0.04
Upeneichthys vlamingii BV	0.264						+		0.01	3	204.05	1.73	0.04
Upeneichthys vlamingii TV	-0.067	+						+	0.1	5	177.95	0	0.11
Upeneichthys vlamingii TV	-0.066	+							0.05	3	178.77	0.82	0.07
Upeneichthys vlamingii TV	-0.076				+				0.04	3	179.23	1.28	0.06

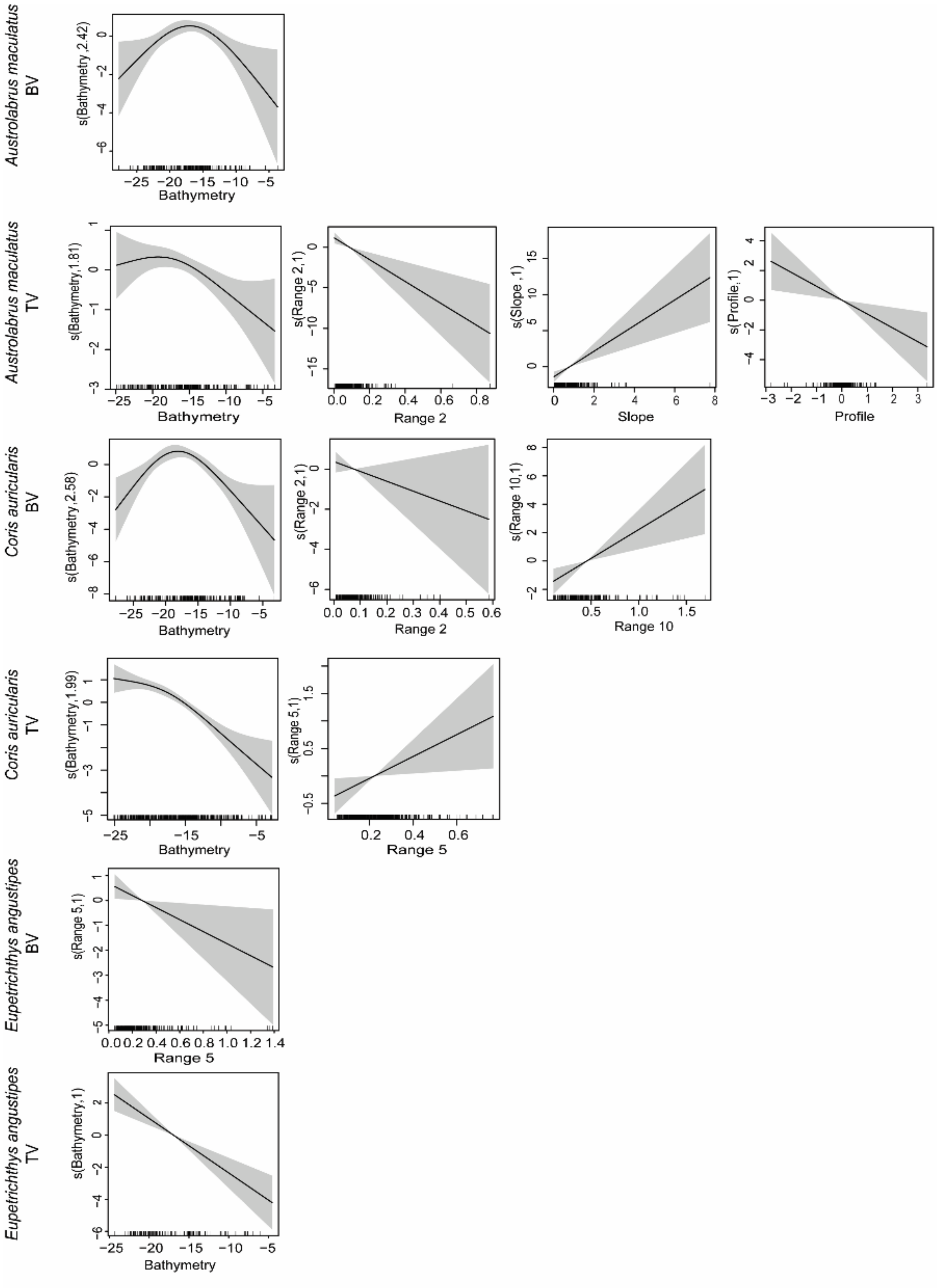

Supplementary Fig. S1. Smoother estimates (solid line) for the environmental predictors as obtained by generalised additive models for six study fish species across two survey methods: baited video (BV) and towed video (TV). The approximate 95% confidence envelopes are indicated (grey shading), marks along the x-axis are sampled data points. All explanatory variables were fitted with model smooths (knots) k = 4. Summary of the environmental predictors is provided in Supplementary Table S3.

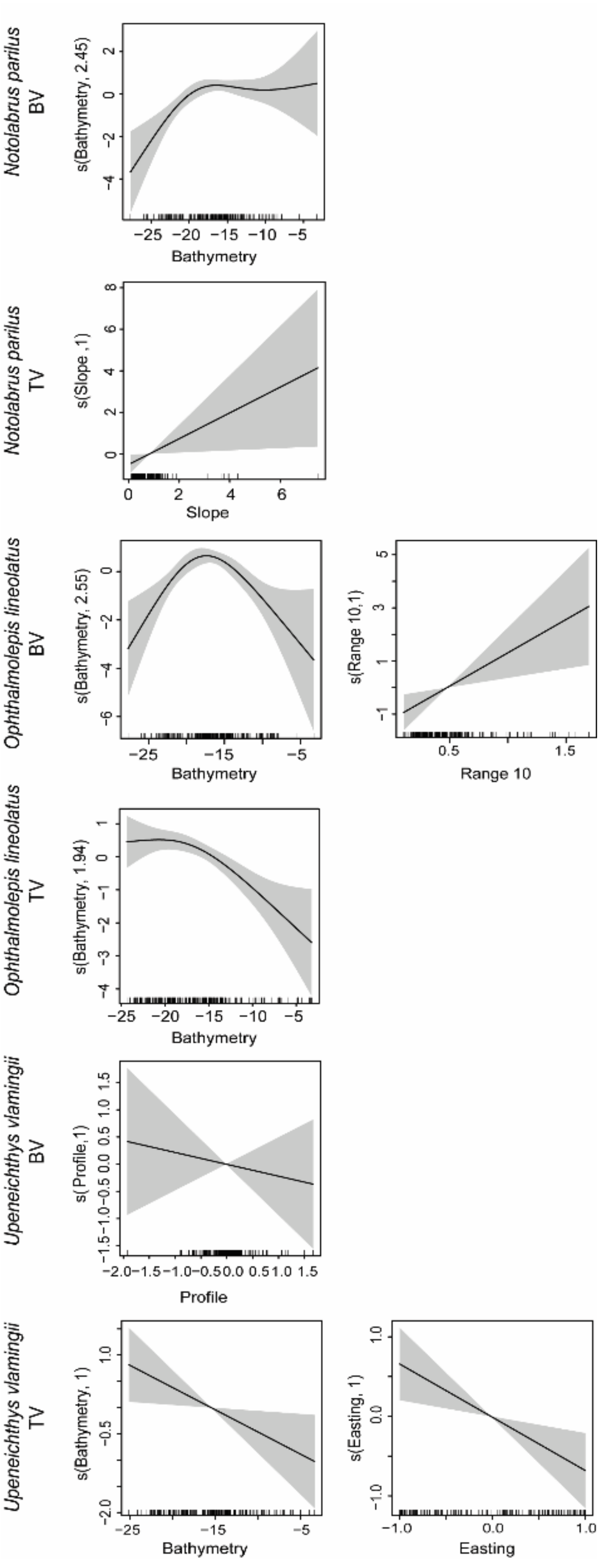

Supplementary Fig. S1 continued. Smoother estimates (solid line) for the environmental predictors as obtained by generalised additive models for six study fish species across two survey methods: baited video (BV) and towed video (TV). The approximate 95% confidence envelopes are indicated (grey shading), marks along the x-axis are sampled data points. All explanatory variables were fitted with model smooths (knots) k = 4. Summary of the environmental predictors is provided in Supplementary Table S3.

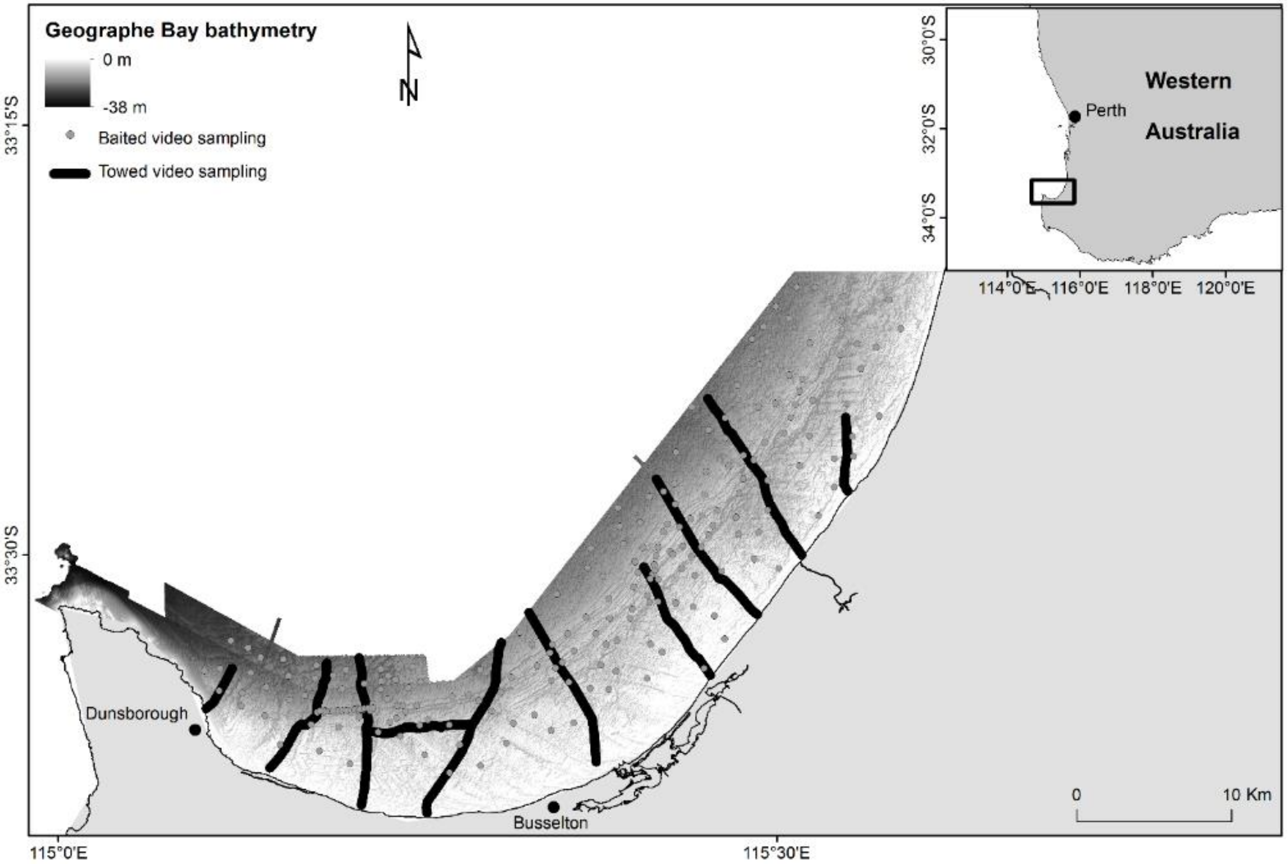

Supplementary Fig. S2. Inset: The location of the Geographe Bay study area on the south-west coast of Western Australia. Main map: Shading indicates bay's bathymetry. Black lines indicate towed video transects. Grey dots indicate baited remote stereo-video deployments. This map was created using ArcGIS version 10.1 (ESRI (2011) ArcGIS Desktop: Release 10.1. Environmental Systems Research Institute, Redlands, CA).

Supplementary Table S2. Fish species modelled, with summary of the number of occurrences used in model building based on the two survey methods: baited video (BV) and towed video (TV).

Scientific name	Common name	Family	Method	Presence	Pseudo-Absence	Additional info
Austrolabrus maculatus	Black-spotted wrasse	Labridae	BV	97	108	Small size endemic species
			TV	117	117	
Coris auricularis	Western King wrasse	Labridae	BV	140	60	Large size mobile endemic species
			TV	234	234	
Eupetrichthys angustipes	Snakeskin wrasse	Labridae	BV	58	155	Small size endemic species
			TV	59	59	
Notolabrus parilus	Brown-spotted wrasse	Labridae	BV	140	65	Large size endemic species
			TV	70	70	
Ophthalmolepis lineolatus	Southern Maori wrasse	Labridae	BV	150	63	Large size mobile endemic species
			TV	113	113	
Upeneichthys vlamingii	Blue-spotted Goatfish	Mullidae	BV	121	86	Mobile species, bycatch in commercial and recreational fisheries
			TV	85	85	

Supplementary Table S3. Description of the seafloor variables used in model building.

Environmental Predictor	Description
Bathymetry	Elevation in metres relative to the Australian Height Datum.
Eastness	Trigonometric transformation of a circular azimuthal direction of the slope (*sin* (aspect)). Values close to 1 represent east-facing slope, close to -1 if the aspect is westward.
Northness	Trigonometric transformation of a circular azimuthal direction of the slope (*cos* (aspect)). Values close to 1 represent north-facing slope, close to -1 if the aspect is southward.
Slope	First derivative of elevation. Average change in elevation, steepness of the terrain, % rise.
Range 2,5,10	Maximum minus the minimum elevation in the local neighbourhood (local relief). Calculated at window sizes of 2*2, 5*5, 10*10 cells respectively, which equates to ground area of 64, 400 and 1600 m2 (i.e. fine, medium and coarse scale local relief).
Plan curvature	Secondary derivative of elevation. Measure of concave/convexity perpendicular to the slope.
Profile curvature	Secondary derivative of elevation. Measure of concave/convexity parallel to the slope.
Curvature	Combined index of profile and plan curvature.
